# The production of fluorescent transgenic trout to study *in vitro *myogenic cell differentiation

**DOI:** 10.1186/1472-6750-10-39

**Published:** 2010-05-17

**Authors:** Jean-Charles Gabillard, Cécile Rallière, Nathalie Sabin, Pierre-Yves Rescan

**Affiliations:** 1 National Institute for Agricultural Research, Joint Research Unit for Fish Physiology, Biodiversity and the Environment, INRA Scribe, IFR140, Campus de Beaulieu, 35042 Rennes, France

## Abstract

**Background:**

Fish skeletal muscle growth involves the activation of a resident myogenic stem cell population, referred to as satellite cells, that can fuse with pre-existing muscle fibers or among themselves to generate a new fiber. In order to monitor the regulation of myogenic cell differentiation and fusion by various extrinsic factors, we generated transgenic trout (*Oncorhynchus mykiss*) carrying a construct containing the green fluorescent protein reporter gene driven by a fast myosin light chain 2 (MlC2f) promoter, and cultivated genetically modified myogenic cells derived from these fish.

**Results:**

In transgenic trout, green fluorescence appeared in fast muscle fibers as early as the somitogenesis stage and persisted throughout life. Using an *in vitro *myogenesis system we observed that satellite cells isolated from the myotomal muscle of transgenic trout expressed GFP about 5 days post-plating as they started to fuse. GFP fluorescence persisted subsequently in myosatellite cell-derived myotubes. Using this *in vitro *myogenesis system, we showed that the rate of muscle cell differentiation was strongly dependent on temperature, one of the most important environmental factors in the muscle growth of poikilotherms.

**Conclusions:**

We produced MLC2f-gfp transgenic trout that exhibited fluorescence in their fast muscle fibers. The culture of muscle cells extracted from these trout enabled the real-time monitoring of myogenic differentiation. This *in vitro *myogenesis system could have numerous applications in fish physiology to evaluate the myogenic activity of circulating growth factors, to test interfering RNA and to assess the myogenic potential of fish mesenchymal stem cells. In ecotoxicology, this system could be useful to assess the impact of environmental factors and marine pollutants on fish muscle growth.

## Background

In teleost fish, muscle growth results from successive phases of myogenesis that involve distinct myogenic precursor populations [1]. During the final and most important phase of myogenesis (termed mosaic hyperplasia) which starts around the larval stage, new muscle fibers are continuously being added throughout the myotome, giving a typical mosaic appearance to a muscle cross section [2]. Thus, unlike mammals, in which postnatal growth is solely dependent on the hypertrophy of muscle fibers formed during the embryonic period, fish combine muscle fiber hypertrophy and hyperplasia to generate indeterminate muscle growth throughout life [3]. The satellite cells that lie under the basal laminae of muscle fibers provide the myonuclei necessary for fiber recruitment and hypertrophy [4]. Satellite cells from a variety of fish species, including trout [5], carp [6], salmon [7] and sea bream [8], have been isolated and cultured *in vitro*. One of the limitations of these cell cultures is that contaminating non-myogenic cells, especially fibroblast-like cells, proliferate extensively as culture progresses, thus hampering the recognition of myogenic cells and monitoring of their differentiation. To enable the real-time monitoring of the progression of cultivated muscle cell differentiation, we thus have generated transgenic trout specifically expressing GFP in muscle fibers and then cultivated genetically-modified muscle-derived cells from these animals.

## Methods

### Animals

All the experiments were carried out on the rainbow trout *Oncorhynchus mykiss *(Walbaum). Transgenic fish and their progenies were maintained at 10°C in recirculating water under artificial light-darkness conditions, mimicking the annual photoperiod variations. The chemical parameters of the water were regularly monitored. Oxygen levels always remained above 98% saturation.

### Transgene construct

**Figure 1:**
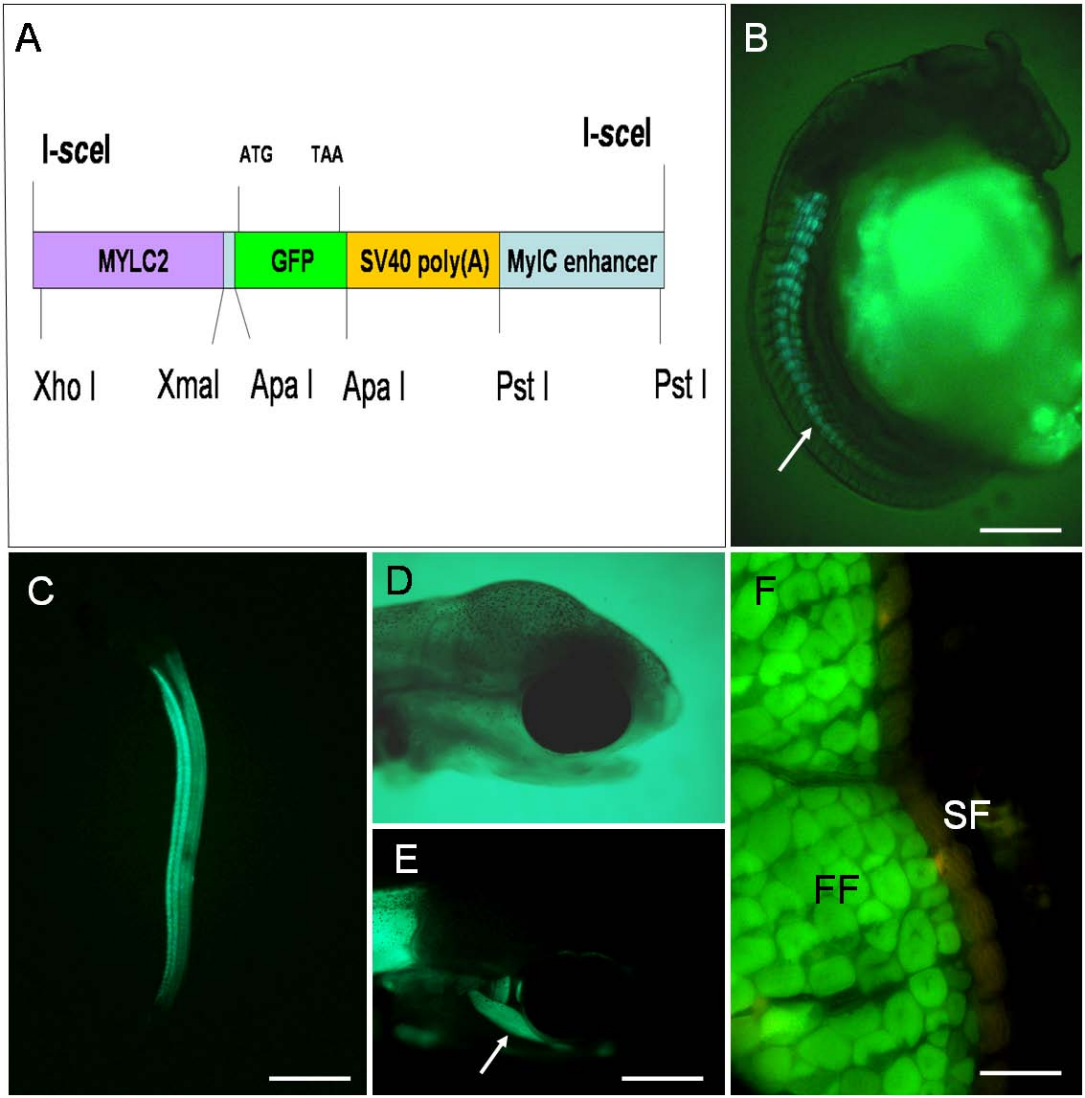
**(A) Structure of the MLC2f-GFP transgene**. This construct contains a myosin light chain (MLC2f) promoter at the 5', the GFP coding region, a fragment including the small t intron and polyadenylation signal from SV40 and a mylc 1/3 enhancer. The restriction sites for ligation are represented. The transgene is flanked by I-sceI sites so that it achieves I-SceI meganuclease-mediated transgenesis. **(B-F) Expression of GFP in transgenic trout**. (**B**) 50 somite stage embryo, lateral view: GFP labeling is present in most rostral somites and initially appears in medial domain of the somite (arrow). (**C-E**) Hatching stage embryo. (**C**) ventro-lateral view: GFP expression is present in myomeric axial musculature, (**D, E**) lateral view, bright field light (**D**) and fluorescence microscopy (**E**): GFP expression is detected in the head muscles (arrow). (**F**) Free swimming larvae. Transverse section: GFP is expressed in deep fast muscle fiber (FF) but not in superficial slow (SF) fibers. Scale bar = 500 μm in B, 2 mm in C, 350 μm in D and E and 40 μm in F.

The fMyLC2-GFP transgene construct used during this study contained a 1 kilobase zebrafish fast myosin light chain 2 (MLC2f) promoter at the 5' [9], the 0.8 kb GFP coding region, a 860pb fragment including the small t intron and the polyadenylation signal from SV40 [10] and a mylc 1/3 enhancer from the rat mylc gene [11] (Figure 1A). All DNA fragments that served for the transgene construct were amplified by PCR using primers containing restriction sites for ligations [see additional file 1]. All subcloning procedures were performed in a modified pBluescriptII SK+ carrying I-SceI sites in both ends of its polylinker to enable I-SceI meganuclease-mediated transgenesis [12].

### Microinjection in trout eggs

Eggs were collected by gentle manual stripping performed under anesthesia (2-phenoxyethanol, 0.05%). DNA construct was microinjected during the period of 2-6 h after fertilization, i.e. after formation of the embryonic disc and prior to the first cleavage, using the two-step method reported by Chourrout et al. [13]. The injected solution contained 30 ng/μl DNA, I-SceI buffer (0.5 ×, New England Biolabs, USA), meganuclease I-SceI (New England Biolabs, USA) at a rate of 1 unit/μl, and 0.1% phenol red.

### Production of transgenic trout and GFP observations

Embryos raised from eggs microinjected with the MlC2f-GFP construct were examined for GFP expression at the hatching stage. GFP-positive F0 embryos were then collected and raised to sexual maturity. Semen from GFP-positive males was used to fertilize wild-type eggs in order to produce F1 offspring. For histological examinations, samples were embedded in 30% ovalbumin, 0.5% gelatine and 1% gluteraldehyde in PBS. Blocks were sectioned at 40 μm on a Leica vibratome. Whole mount and transverse section images were obtained using a Nikon MULTIZOOM AZ100 microscope and a Nikon ECLIPSE 90i microscope, respectively.

### Isolation and culture of satellite cells

Satellite cells were dissected, isolated and cultivated according to the procedure described by Rescan et al. [14]. Briefly, white epaxial muscle from 5-10 g F1 trangenic trout was excised under sterile conditions and collected in DMEM (Gibco) medium containing penicillin (100 U/ml), streptomycin (100 μg/ml), amphotericin B (0.25 μg/ml) and gentamicin (75 μg/ml) The tissue was minced into small pieces, centrifuged and treated for 1 h with a 0.2% collagenase solution. After centrifugation, the pellet was resuspended twice in a 0.1% trypsin solution for 20 min. The fragments were then dissociated mechanically and the resulting suspension was filtered through a 20 μm filter mesh. The cells thus collected were seeded on a laminin layer which was deposited on a pre-coated poly-lysine substratum [6]. The muscle cell extract was left on the substrate for 30 minute and non adherent cells were removed by washing. Remaining cells were cultured in DMEM medium that was continuously supplemented with 10% calf serum and antibiotics. The cells were incubated at either 18°C or 10°C immediately upon seeding of the plates. Cell culture images were obtained on a Nikon ECLIPSE 90i microscope.

## Results

### Establishment of stable transgenic trout strains

Transgenesis was performed in trout. We have used trout because it is a worldwide commercial species that display a continuous muscle growth strongly powered by hyperlastic process [2]. Also this species which grow to a large final size is suitable for obtaining large amount of myogenic cells for culture purposes [14]. After injection of the plasmid with *I-SceI *enzyme, about 60% of the surviving hatching embryos displayed muscle fluorescence. GFP expression was markedly mosaic, the number of GFP-positive myofibers ranging from a few fibers to an almost ubiquitous labeling of muscle myofibers. Six of the most fluorescent GFP-positive embryos were raised to sexual maturity and outcrossed to the wild-type strain. Three founder fish (50%) were thus obtained, as shown by the presence of non-mosaic GFP expression in F1 offspring.

### GFP expression in developing and adult F1 transgenic trout

GFP expression in F1 offspring was first detectable at around the 40-somite stage in the most rostral somites. GFP fluorescence subsequently progressed caudally as somites formed in a rostral-to-caudal wave (Figure 1, B and 1C). During somite maturation, GFP expression progressed medio-laterally, starting deep in the somite and then extending to more ventral and dorsal regions (Figure 1B). At the end of somitogenesis, GFP fluorescence was observed within the whole myomeric axial musculature (Figure 1C) as well as in eye and jaw muscles (Figure 1D and 1E). After development, fluorescence persisted throughout the life of the transgenic trout in the deep fast muscle fibers making up the major part of the myotomal musculature, but was excluded from slow muscle fibers at the periphery of the myotome (Figure 1F).

### GFP expression in cultured myosatellite cells

**Figure 2:**
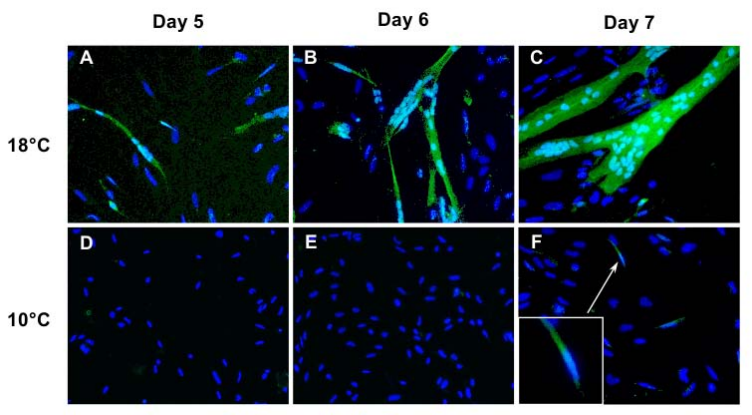
**GFP expression in genetically-modified trout muscle cells**. Muscle cells cultivated at 18°C (A-C) or 10°C (D-F), 5 (A and D), 6 (B and E) and 7 days (C and F) post-seeding. At 18°C, GFP is visualized in fusing myogenic cells (day 5) and then in large myotubes (day 6 and 7). At 10°C, there is no GFP positive cell at day 5 and 6 of culture and only small myotubes express GFP at day 7. Nuclei were stained with Hoechst.

Myosatellite cells were isolated from the fast muscle of juvenile transgenic trout and then cultivated on a laminin substrate. The cells lined up about four days after plating and displayed GFP fluorescence on day 5 (Figure 2A). The first fusion occurred at this time (Figure 2A), leading to large GFP-positive multinucleated myotubes at day 6 and 7 (Figure 2B and 2C). Numerous cells that did not express GFP were also observed in the vicinity of the myotubes, suggesting that non-myogenic cells were seeded during the establishment of primary cultures (Figure 2B and 2C). Overall, these observations showed that cultivated muscle cells from the genetically-modified trout expressed GFP while they exhibited morphological features of differentiation.

### Culture of genetically-modified myogenic cells showed that temperature influenced the rate of muscle cell differentiation

**Figure 3:**
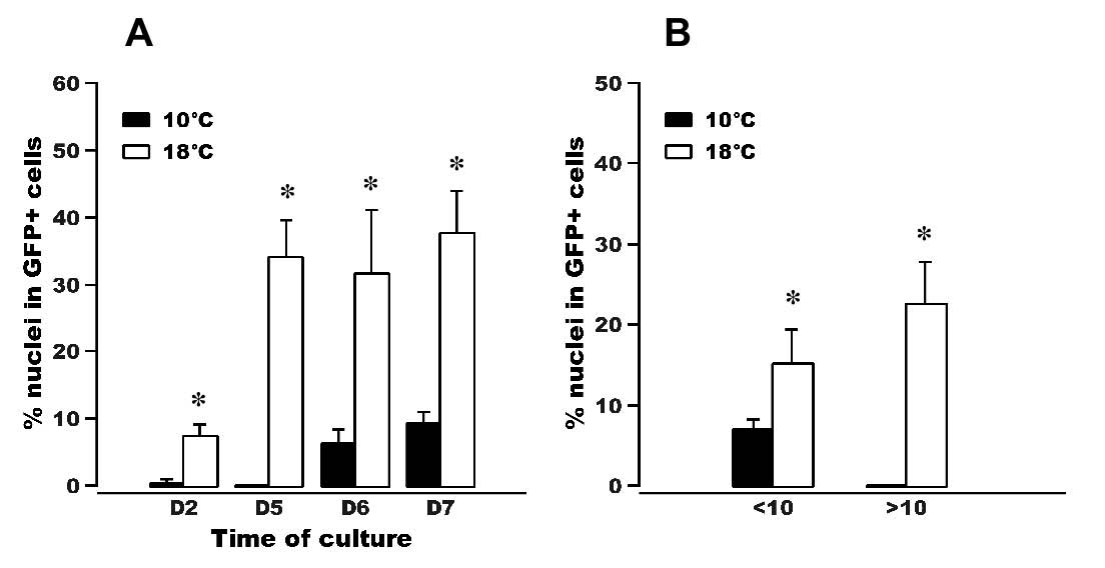
**Temperature influences the rate of muscle cell differentiation**. (A) Percentage of nuclei contained in GFP-positive cell structures. The muscle cells were cultured at either 10°C or 18°C. The proportion of nuclei in GFP-positive cells was determined 2, 5, 6 and 7 days post plating. (B) Percentage of nuclei contained in small multinucleated myotubes (less than 10 nuclei) or in large multinucleated syncytia (more than 10 nuclei), 7 days after plating. Stars indicate significantly different means at P > 0.05.

In order to assess the ability of our in vitro myogenesis system to investigate direct effect of extrinsic factors on muscle cell differentiation and growth we isolated muscle cells from the transgenic trout and cultivated them at two temperatures: 10°C and 18°C. Contrary to what was observed at 18°C (see above), no GFP expression could be detected in muscle cells cultivated at 10°C five and six days after plating (Figure 2D and 2E). At day 7, few nuclei were found to be contained in GFP-positive cells (Figure 2F and Figure 3A and 3B) while numerous nuclei were incorporated in branched cellular networks of myotubes that were formed at 18°C (Figure 2C and Figure 3A and 3B). On the whole use of our in vitro myogenesis system with GFP cells thus showed that temperature, one of the most important environmental factors affecting the growth of poikilotherms, exerted a pronounced and direct effect on the differentiation, fusion and growth of cultivated satellite cells.

## Discussion

The differentiation of myogenic cells, and their fusion into multinucleated myotubes, is regulated by a variety of extrinsic physical and chemical factors. Identification of these factors can be facilitated by muscle cell cultures, and in this regard the use of mouse myogenic cell lines such as C2C12 [15] has been particularly fruitful. Unfortunately, such cell lines are limited to a narrow range of species, and *in vitro *studies on muscle cells from domestic animals and lower vertebrates - particularly fish - can only be performed using primary cultures [16]. Because of the presence of contaminating non-myogenic cells in fish primary myoblast cultures, it is often difficult to recognize myogenic cells and monitor their differentiation without applying the immunolocalization of muscle markers. In an attempt to circumvent this problem, we generated transgenic trout specifically expressing GFP in differentiated muscle cells and cultivated muscle-derived cells from these animals.

To generate the transgenic trout we adopted the meganuclease mediated transgenesis method [12]. This approach has been reported to increase in medaka fish the number of individuals expressing a transgene, to decrease the level of mosaicism and to enhance the frequency of transgenic F1 offspring [12]. The meganuclease protocol has been successfully used in various animals such as Xenopus, sea urchin and sea anemone [17-20]. The mechanisms underlying the generally high frequencies of transgenesis obtained with this method are unclear. One possible explanation is that the continued stable binding of the enzyme to the DNA ends prevents concatemer formation and/or degradation of linear monomers, which generally inhibits integration of transgenes into the host genome. The *mylc *1/3 enhancer from the rat *mylc *gene was added to the transgene vector because this sequence has been reported to increase muscle-specific promoter activity in mouse [11] as well as in zebrafish [21]. However, we recently found that the deletion of this enhancer had no apparent effect on the muscle-specificity and activity of the MLC2f promoter showing that the MLC2f promoter contained the essential regulatory sequence for strong muscle-specific expression.

In the genetically modified trout, the expression pattern of the MLC2f-GFP transgene mimicked the expression of the endogenous MLC2f gene [9]. Indeed, GFP fluorescence in transgenic embryo swept from the anterior to the posterior, progressed mediolaterally within the maturing somite and, in late stage of development, was also detected in muscles of the head. As expected from the *in vivo *GFP gene expression pattern, isolated and cultured muscle cells derived from the transgenic trout displayed GFP expression that was concomitant with morphological features of differentiation such as cell fusion and myotube growth. Thus our *in vitro *myogenesis system was able to directly monitor the evolution of muscle cell differentiation and would therefore be quite convenient to assess the effects of various extrinsic factors on muscle differentiation and growth. In this regard, use of our system showed that a lower temperature led to a decrease in the differentiation rate, as previously shown in the salmon [7]. This result obtained *in vitro *was consistent with those reported *in vivo *which showed that the growth rate of trout muscle increases in line with temperature when it ranges from 5°C to 15°C [22]. Beside testing environmental parameters this system could be used to assess the myogenic effects of different endocrine and circulating factors [23], and the myogenic function of individual genes using small interfering RNA (siRNA) [24].

Until recently, the muscle satellite cells situated between the basal lamina and cell membrane of mature myofibers [25] were presumed to be the only source of myogenic cells for muscle growth and repair. However, pluripotent stem cells that can be isolated from mesenchymal tissues [26,27] and from several other tissues [28,29] now constitute alternative sources of myogenic cells. It has been shown in the mouse that mesenchymal stem cells from adipose tissue can spontaneously differentiate into skeletal muscle, and that their conversion to a myogenic phenotype is enhanced by co-culture with primary myoblasts [27]. Non-muscle tissues from GFP transgenic trout would be particularly useful for similar studies in fish, insofar as the appearance of any green fluorescence in cultures, or in co-cultures mixing mesenchymal cells from GFP trout and wild-type (GFP-negative) myoblasts, would indicate the incorporation of non-muscle cells into skeletal myotubes.

## Conclusion

We have produced genetically-modified trout harboring a MLC2f-GFP transgene that is active in fast muscle fibers. Muscle cells derived from these transgenic trout became fluorescent *in vitro *when they differentiate. The *in vitro *myogenesis system thus developed enables the real-time monitoring of muscle differentiation and growth in order to dissect the physiology of fish muscle cells. Using this system, we have shown that temperature, an important environmental factor for poikilotherms, influences the rate of differentiation of trout myogenic cells.

## Authors' contributions

PYR conceived the study and wrote the paper. CR and PYR constructed the transgene vector. PYR produced the transgenic trout. JC and NS extracted and cultivated muscle cells from transgenic animals and performed *in vitro *observations. All authors read and approved the manuscript.

## Supplementary Material

Additional file 1 **Primers used for transgene construction**. Additional file 1 contains primers used for generating DNA fragments that served for the transgene construction. The restriction sites for ligations are underlined. Click here for file
