# Supplementary material for: The production of fluorescent transgenic trout to study in vitro myogenic cell differentiation
Source: BMC Biotechnol. 2010 May 17;10:39. doi: 10.1186/1472-6750-10-39 (PMC2887378; doi:10.1186/1472-6750-10-39)
Supplement: Additional file 1 — Primers used for transgene construction. Additional file 1 contains primers used for generating DNA fragments that served for the transgene construction. The restriction sites for ligations are underlined. [file 1472-6750-10-39-S1.DOC]

**Myosin light chain 2 (MLC2f) promoter:**

F: ATTGCACTCGAGTAGTCTAAAGAAAGAAGGGAATG

Xho I

R : ATTGCACCCGGGGTGTGAAGTCTAAGAAGATCA

XmaI

**GFP cDNA:**

F: TTGCAGGGCCCCTGATCCACCGGTCGCCACCATGGT

Apa I

R: TTGCAGGGCCCGCCGCTTTACTTGTACAGCTCGTC

Apa I

**Polyadenylation signal from SV40**

F: TTGCAGGGCCCTTTGTGAAGGAACCTTACTTC

Apa I

R: TTGCACCTGCAGCCAGACATGATAAGATACATT

Pst I

**Mylc 1/3 enhancer**

F: TTGCACTGCAGGCTATTAATCCCAGAGCCCTTG

Pst I

R: TTGCACTGCAGGCTTTAAGATACAATTTATTTTTTC

Pst I
